# Beat-to-beat analysis of hemodynamic response to mental and psychological stress in sickle cell anemia

**DOI:** 10.1093/jscdis/yoae010

**Published:** 2024-10-28

**Authors:** Arash Abiri, Sara Marmarchinia, Payal Shah, Wanwara Thuptimdang, Thomas D Coates, Michael C K Khoo, Michelle Khine

**Affiliations:** Department of Biomedical Engineering, University of California Irvine, Irvine, CA, 92697, United States; Department of Biomedical Engineering, University of California Irvine, Irvine, CA, 92697, United States; Division of Hematology, Children’s Center for Cancer and Blood Diseases, Children’s Hospital Los Angeles, Keck School of Medicine, University of Southern California, Los Angeles, CA, 90027, United States; Department of Biomedical Sciences and Biomedical Engineering, Faculty of Medicine, Prince of Songkla University, Hat Yai, Songkhla, 90110, Thailand; Division of Hematology, Children’s Center for Cancer and Blood Diseases, Children’s Hospital Los Angeles, Keck School of Medicine, University of Southern California, Los Angeles, CA, 90027, United States; Department of Biomedical Engineering, Keck School of Medicine, University of Southern California, Los Angeles, CA, 90007, United States; Department of Biomedical Engineering, University of California Irvine, Irvine, CA, 92697, United States

**Keywords:** SCD, SCA, blood pressure variability, beat-to-beat blood pressure, stress, vaso-occlusive crises

## Abstract

**Objectives:**

Vaso-occlusive crises are a hallmark symptom of SCD. Physical stressors can trigger decreased microvascular blood flow and increase the risk for vaso-occlusive crises. However, the effect of mental and psychological stressors on vascular physiology in SCD is not well-established. We hereby examined fluctuations in continuous blood pressure to evaluate hemodynamic changes in SCD patients during mental and psychological stress.

**Methods:**

Thirteen SCD subjects from the Children’s Hospital Los Angeles and 11 healthy volunteers were recruited. Continuous blood pressure was recorded during 2 mental tasks and 1 psychological stress task. Systolic beat-to-beat blood pressure variability measurements were calculated for each subject. Three very short-term blood pressure variability metrics served as outcome measures: SD, coefficient of variation, and average real variability. Peripheral augmentation index was calculated from arterial waveforms. Linear mixed effects models evaluated associations between patient factors and outcome measures.

**Results:**

SCD patients exhibit increased systolic blood pressure variability in response to psychological stress. All subjects exhibited a decrease in systolic blood pressure variability in response to mental stress tasks. During mental stress, both groups displayed increased augmentation index, reflective of stress-induced vasoconstriction, while psychological stress in SCD patients led to both decreased mean arterial pressure and increased augmentation index, suggestive of uncompensated vasoconstriction.

**Conclusion:**

These findings emphasize the impact of mental and psychological stressors on vascular function in SCD and the potential for monitoring physiological signals to predict vaso-occlusive crisis events.

## Lay Summary

This study explores how both mental and psychological stress affects people with SCD, a condition known for causing severe pain episodes called vaso-occlusive crises. Traditionally, physical stressors were known to trigger these painful episodes by reducing blood flow in small blood vessels. However, the impact of mental or psychological stress on these patients is not yet fully understood. To investigate, we tracked continuous blood pressure in SCD patients and healthy subjects during tasks designed to induce mental and psychological stress. We found that SCD patients showed higher variations in systolic blood pressure, especially under psychological stress, indicating that their blood vessels were more likely to constrict abnormally. Both groups similarly experienced blood vessel constriction during mental stress tasks. These results suggest that mental and psychological stress can significantly affect vascular function in SCD patients, potentially leading to painful episodes of vaso-occlusive crises. Therefore, this study highlights the importance of stress management in preventing vaso-occlusive crises and the potential to reduce these episodes by implementing proactive interventions in response to abnormal blood pressure variations.

## INTRODUCTION

SCD is a genetic disorder affecting millions of people globally. It is caused by an amino acid alteration in the Hb β chain that produces abnormal HbS. Under conditions of reduced oxygen levels, the presence of HbS leads to the formation of sickle-shaped red blood cells, which are prone to clumping together, obstructing blood vessels, and impeding blood flow.^1^ This painful vaso-occlusive crisis (VOC) is a hallmark symptom of SCD and triggers a cascade of complications, including tissue ischemia, chronic anemia, and endothelial dysfunction, ultimately contributing to the multifaceted manifestations of the disease.^1^^,^^2^ Various demographic and clinical factors, such as age, fetal Hb concentration, and blood viscosity, have been linked with how frequently VOCs occur.^3–5^ Given that painful VOCs can be unexpectedly triggered by stressful events, there is a growing interest in elucidating the pathophysiological mechanisms responsible for this immediate transition from steady state to VOC.^6^

Given the well-established role of stress in activating the autonomic nervous system (ANS),^7^^,^^8^ which is a major regulator of precapillary blood flow, it is hypothesized that ANS dysfunction in SCD can impair blood flow responses to stressors and increase the transit time of erythrocytes in the microvasculature, thereby putting SCD individuals at higher risk of having VOCs. A variety of physical and mental stressors have been previously implicated in altering ANS modulation and worsening SCD.^9^ Studies exposing SCD individuals to hypoxia, cold face stimulation, heat pain, and orthostatic stress have demonstrated significant deviations in ANS responses compared to healthy subjects.^10–13^ Psychological factors, such as perceived mood and anxiety levels, have been reported to influence SCD patients’ quality of life as well as the severity and frequency of pain events.^14–16^ However, there is a paucity of studies examining the underlying pathophysiology involved in psychological stress. Recent studies on mental stress (MTS) have attempted to elucidate this phenomenon by introducing cognitive tasks to SCD individuals and examining changes in microvascular blood flow and baroreflex activity.^6^^,^^17^ Although SCD patients were found to exhibit a lower baseline baroreflex activity than healthy subjects, no discernible differences in vasoconstriction were found. Therefore, it is not yet clear what unique physiological changes occur in SCD patients in response to mental and psychological stressors that may contribute to VOCs.

In this study, we aimed to fill this gap in knowledge by using pulse wave analysis (PWA) to evaluate hemodynamic changes from continuous blood pressure (BP) signals in response to mental and psychological stress tasks in SCD and healthy subjects.^18^ Continuous BP offers valuable insights into both autonomic function and vascular tone by providing high-resolution data on blood flow dynamics. We applied PWA on continuous BP signals to quantify beat-to-beat BP variability (BPV), mean arterial pressure (MAP), and peripheral augmentation index (AI). Beat-to-beat fluctuations in BP arise from the intricate interplay of hemodynamic factors, neuronal reflexes, hormonal, behavioral, and environmental stimuli.^19^ Increased variability in beat-to-beat BP implicates ANS disturbance and baroreflex dysfunction.^20^ Peripheral AI, commonly used as a measure of arterial stiffness at steady state, serves as an indicator of vasoconstriction over short measurement periods.^21–24^ By analyzing concomitant changes in AI and MAP, we could obtain unique perspectives on autoregulatory function in SCD patients. Thus, we sought to use beat-to-beat BPV, MAP, and AI to identify differences in how SCD individuals physiologically respond to mental and psychological stress compared to healthy subjects. Elucidating these physiological differences will lay the framework for developing predictive algorithms to prevent painful events as well as contribute to evidence-based guidelines on SCD patient counseling and management.

## MATERIALS AND METHODS

### Participants

This study was conducted at the Children’s Hospital Los Angeles (CHLA) in accordance with an institutional review board-approved protocol. Informed consent/assent was obtained from all study subjects. Participants were eligible if they were 13 years of age or older and competent to understand consent and protocol instructions. Subjects were excluded if they had underlying anxiety disorders, pain crises, or were hospitalized within 10 days of study participation. Subjects were also excluded if they had any known acute or chronic illnesses that could compromise subject safety or data integrity. Thirteen SCD patients with homozygous sickle Hb (HbSS) who were stable either on chronic transfusion or hydroxyurea were recruited from the hemoglobinopathy center at CHLA. Eleven non-SCD (control) individuals with normal Hb (HbAA) were recruited from patients’ families and friends to serve as race-matched controls.

### Experimental protocol

All experiments were performed in an ANS laboratory under strictly controlled settings.^13^ Measurements were carried out in a quiet, dimly lit, temperature-controlled room. Each subject rested comfortably at an approximately 45-degree angle on a cushioned chair with arm and leg supports. Subjects were instructed to rest quietly for 15 minutes to allow all signals to stabilize (after monitoring devices were connected) before any recordings began.

The State-Trait Anxiety Inventory (STAI) Y-1 and Y-2 questionnaires were administered at baseline to evaluate *state anxiety* (ie, anxiety at the moment) and *trait anxiety* (ie, how they generally feel), respectively.^25^ Five minutes of beat-to-beat BP measurements were subsequently recorded at baseline, free of any movements.

**Figure 1.**
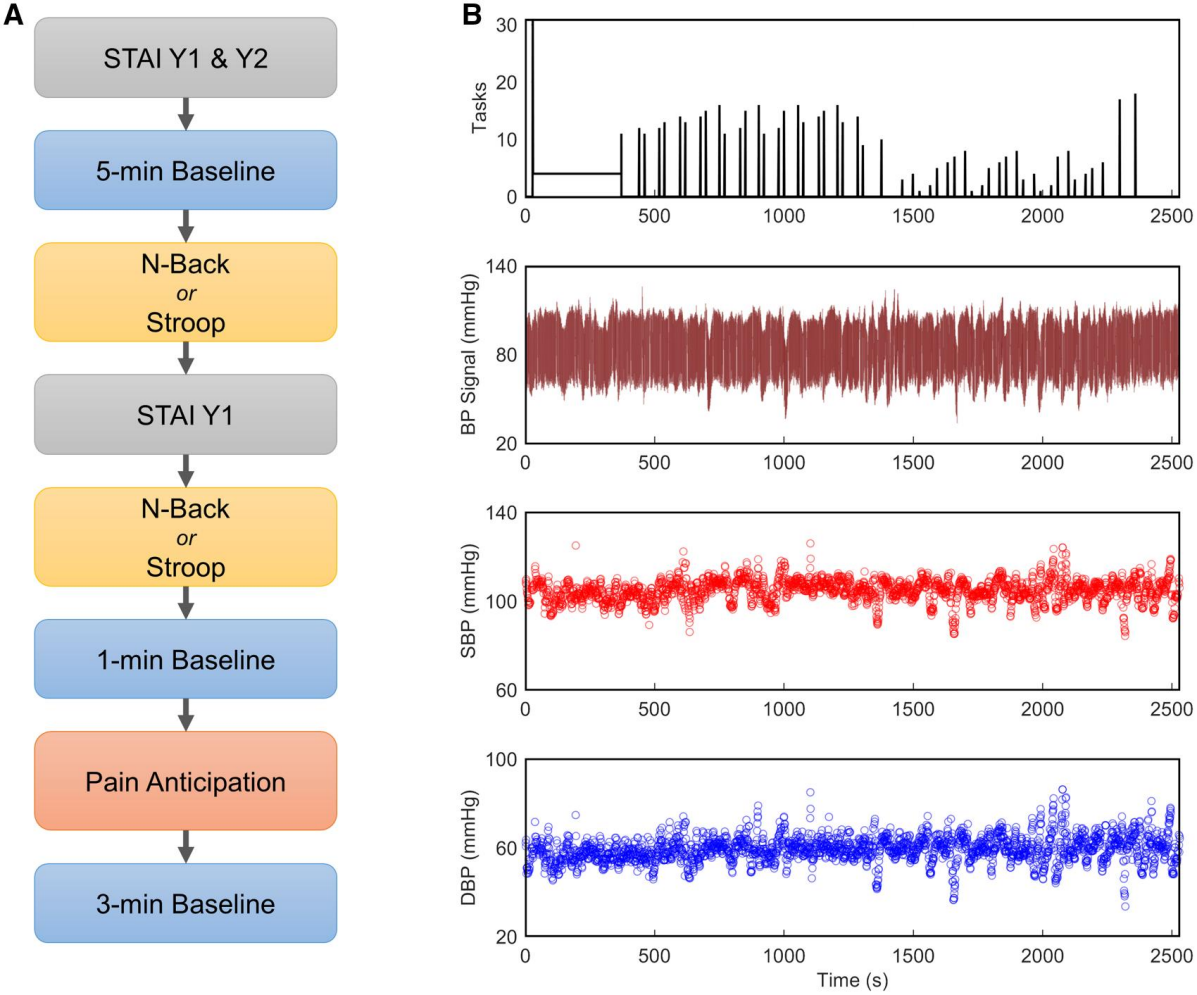
(**A**) Sequence of the study protocol. Subjects were randomly assigned to perform the N-back or Stroop task first. The State-Trait Anxiety Inventory (STAI) Y-1 and Y-2 questionnaires were administered at baseline to evaluate *state anxiety* and *trait anxiety*. Five minutes of beat-to-beat BP measurements were subsequently recorded at baseline. Stress-induction protocol comprised 2 mental stress (MTS) exercises—a memory task (N-back) and a conflict task (Stroop)—presented to subjects in a randomized order, followed by a pain anticipation (PA) task. A follow-up STAI Y-1 questionnaire was administered after the first MTS task to evaluate changes in state anxiety. A 25-second break is placed between N-back sequences. A 20-second break is placed between Stroop sequences. The PA task involved informing subjects that they were going to receive a maximum pain stimulus in 1 minute that would only cease when they could no longer tolerate it and asked for it to stop. (**B**) Example recording panel from a single subject. Top panel represents the output of the E-prime software, where each bar represents task sections and its height representing task difficulty. Raw waveform (second panel) and discrete systolic and diastolic blood pressure (third and fourth panels) signals were collected during the study using the Biopac System. BP: blood pressure; DBP: diastolic blood pressure; SBP: systolic blood pressure.

Following baseline measurements, the stress-induction protocol was initiated using psychological software (E-prime 2.0, Psychology Software Tools, United States), as detailed by Shah et al.^17^ This protocol comprised 2 MTS exercises—a memory task (N-back)^26^ and a conflict task (Stroop)^27^^,^^28^—presented to subjects in a randomized order, followed by a pain anticipation (PA) task (Figure 1A). A follow-up STAI Y-1 questionnaire was administered after the first MTS task to evaluate changes in state anxiety. The N-back task consisted of 12 sequences of alphabetic letters, for each of which subjects were asked to report when a letter was repeated from *n* steps (*n *=* *0, 1, 2, 3) earlier in the sequence. A 25-second break is placed between N-back sequences. The Stroop task consisted of 12 sequences of color words (eg, “blue,” “red”), for each of which subjects were asked to respond by identifying the font color of the color word rather than the written name of the word. A 20-second break is placed between Stroop sequences. The PA task involved informing subjects that they were going to receive a maximum pain stimulus in 1 minute that would only cease when they could no longer tolerate it and asked for it to stop. However, no physical pain was actually administered.

### BP monitoring and analysis

Physiological monitoring sensors were attached to the subject’s left arm. All data were streamed to and synchronized by the Biopac MP150 data acquisition system (Biopac, United States) (Figure 1B). Continuous BP signals were measured at 250 Hz from the left middle finger using a noninvasive finger cuff (Nexfin; BMEYE, Amsterdam, Netherlands). Recordings were exported for processing and analysis in MATLAB (R2022b, The MathWorks, Natick, Massachusetts, United States).

The *BP_annotate* package in MATLAB was used to identify the peaks and troughs in the raw BP signal and to extract each BP waveform.^29^ For each cardiac cycle, the systolic (SBP) and diastolic blood pressure (DBP) were defined as the waveform’s peak and trough, respectively. Mean arterial pressure was calculated for each pulse wave by dividing the area under the BP waveform by the cardiac cycle duration.^30^ Peripheral AI, reported as a percentage, was calculated for each pulse wave by dividing the difference between the late systolic peak and DBP of the arterial waveform by the pulse pressure (Figure S1).^31^ Previous studies have suggested that the AI derived from pulse arterial tonometry is comparable to that derived from synthesized aortic pressure waves (generated using a generalized transfer function on peripheral BP waveforms).^32–36^ AI was used as an indirect measure of peripheral arterial stiffness and vasoconstriction.^21–24^

Multiple beat-to-beat BPV measurements were made for each subject by splitting the entire BP recording into 30-beat segments, which each contained 30 BP values to calculate a BPV value. Three very short-term systolic BPV metrics were calculated: SD, Coefficient of Variation (COV), and Average Real Variability (ARV).^37^ SD is one of the most common variability indices and characterizes the global fluctuation of BP measurements around the mean.^38^ COV is a normalized index of the SD, calculated by dividing the SD by the mean BP.^39^ ARV takes into account the temporal order of BP values and is defined as the average of the absolute differences between adjacent BP measurements.^39–41^

### Statistical analysis

All statistical analyses were performed using R (version 4.0.2; The R Foundation for Statistical Computing) in RStudio (version 2023.09.1). A *P*-value of <.05 was considered statistically significant. Continuous variables were summarized as the mean ± SD. Differences in characteristics between SCD and control patients were assessed using Wilcoxon rank sum tests for continuous variables and Fisher’s exact test for categorical variables. Since multiple measurements were collected per patient for the primary outcome variables (BPV, MAP, AI), baseline differences in means between control and SCD groups were assessed using repeated measures Analysis of Variance. Additionally, linear mixed effects models were used to evaluate associations between patient factors and outcome measures. Normality of residuals was assessed through visualization of QQ-normal plots.^42^ To obtain regression coefficients (β) and 95% CIs from our models, we conducted parametric bootstrapping with 1000 simulations, which mitigated errors in CI calculation in models where residuals were not normally distributed.^43^^,^^44^ Linearity, normality of random effects, homoscedasticity, and multicollinearity were also assessed using the “performance” R package.^45^ Univariate models were first generated using patient characteristics (age, sex, STAI Y-1 and Y-2 scores) and labs (Hb, free plasma Hb, and lactate dehydrogenase levels) as independent variables. Multivariable linear mixed effects models were subsequently generated to identify the association between SCD and outcome measures. Independent variables with *P*-values of <.1 on the aforementioned univariate models or those considered clinically significant (eg, age) based on a priori causal knowledge were included as covariates in the multivariable models.

## RESULTS

BP data were collected from 13 SCD patients and 11 controls. Subject characteristics from both groups are listed in Table 1. At the time of study participation, all SCD patients were anemic and, on average, exhibited a lower Hb than control subjects (*P *<* *.001). Lactate dehydrogenase levels were significantly higher in SCD patients (*P *=* *.003). At baseline, SCD patients exhibited lower beat-to-beat MAP (*P *<* *.001) and AI (*P *<* *.001) than control subjects, but similar systolic BPV (*P *=* *.820). Other demographic factors and baseline characteristics, such as initial BP and STAY Y-1 and Y-2 scores, were similar between the 2 groups (all *P *>* *.05).

**Table 1.** Characteristics of control and SCD patients.

	Control (*N* = 11)	SCD (*N* = 13)	*P*
Hb phenotype	AA	SS	
Age (years)	21.0 [15.0-23.0]	22.0 [16.0-28.0]	.423
Sex, *n* (%)			
Male	5 (45)	7 (54)	>.999
Female	6 (55)	6 (46)	
Hb (g/dL)	12.1 [11.9-13.4]	9.5 [8.6-10.4]	**<.001**
Plasma Hb (g/dL)	11.0 [7.8-16.0]	16.8 [8.1-22.5]	.518
LDH (U/L)	454.0 [415.5-617.0]	797.0 [692.0-1068.0]	**.003**
HR (beats/min)	62.4 [56.6-87.6]	77.4 [69.6-84.5]	.325
SBP (mmHg)	115.7 [107.1-130.5]	115.7 [99.4-130.5]	.908
DBP (mmHg)	77.1 [69.0-85.9]	71.7 [62.4-79.9]	.224
MAP (mmHg)	93.7 [84.3-105.6]	87.0 [79.3-97.6]	**<.001**
AI (%)	49.4 [41.5-62.5]	46.4 [37.4-57.4]	**<.001**
SBPV, SD (mmHg)	4.0 [2.5-5.6]	4.2 [3.1-5.1]	.820
State anxiety	27.0 [22.5-32.5]	28.0 [23.0-34.0]	.684
Trait anxiety	32.0 [29.0-37.5]	30.0 [26.0-35.0]	.663
Post-exercise anxiety	32.0 [31.0-33.5]	33.0 [26.0-46.0]	.977
Δ State anxiety	4.0 [0.0-7.5]	3.0 [0.0-6.0]	>.999

Abbreviations: AI: augmentation index; DBP: diastolic blood pressure; HR: heart rate; MAP: mean arterial pressure; LDH; lactate dehydrogenase; SBP: systolic blood pressure; SBPV: systolic blood pressure variability.

Continuous variables are reported as median [interquartile range]. Bold indicates statistically significant, *P *<* *.05.

### Changes in BPV

**Figure 2.**
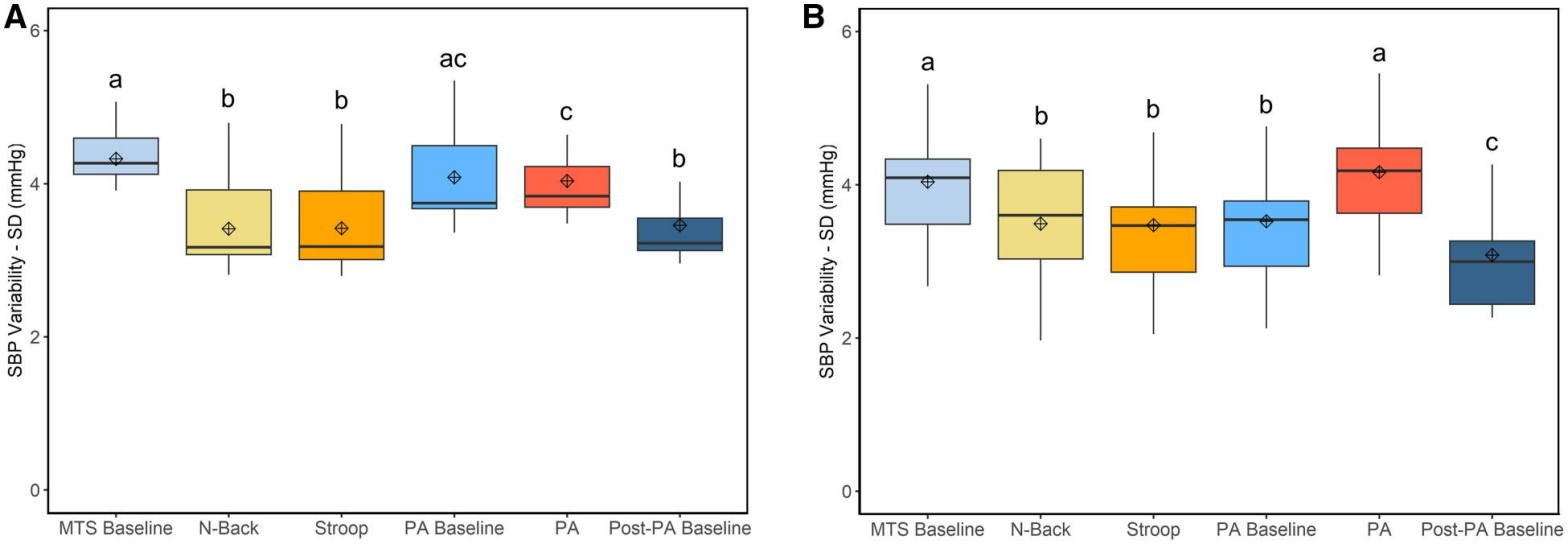
Covariate-adjusted box and whisker plot of the SBP variability at baseline and during study exercises for (**A**) control and (**B**) SCD patients. Significant decreases in SBP variability occurred during N-back and Stroop tasks compared to baseline. Control patients returned to baseline levels following mental stress tasks, but SCD patients did not. An increase in SBP variability occurred during pain anticipation in SCD patients. Diamonds indicate adjusted mean values; horizontal lines indicate adjusted median values. Boxes labeled with different letters indicate a statistically significant (*P* < .05) difference in means. For example, the means for N-back and Stroop were not statistically different from each other in both the subject groups, but were statistically different from their MTS baseline’s mean. MTS: mental stress; PA: pain anticipation; SBP: systolic blood pressure.

On linear mixed effects regression (β = 0.43; 95% CI, 0.20-0.66) and correlation analysis (Figure S2), Hb levels were found to be associated with systolic BPV. In contrast, LDH level was not associated with systolic BPV on regression (*P* = .250) and correlation analysis (Figure S3). Thus, we included Hb levels, SCD state, and mental task type as covariates in our multivariable models. Additionally, we included age as a covariate based on its prior associations with BPV.^46^ When subjects were stratified by SCD state and task type, multivariable linear mixed-effects regression demonstrated that both SCD and control subjects exhibited significantly lower SBP SD during N-back and Stroop tasks compared to baseline (Figure 2, all *P *<* *.05). Following N-back and Stroop tasks, systolic BPV returned back to baseline levels for control subjects, but the BPV of SCD patients remained depressed. Additionally, in contrast to controls, SCD patients exhibited a significantly higher SBP SD during PA compared to the PA baseline (*P *<* *.05).

To better understand the general effect of SCD state on BPV, we also conducted multivariable linear mixed-effects regressions on the entire study population using SBP SD, COV, and ARV as outcome variables and including SCD state and task type as additional independent covariates (Table 2). Mental stress tasks continued to be associated with decreased BPV compared to baseline (all *P* < 0.05). Notably, when considering these effects of stress tasks on BPV, the multivariable models demonstrated SCD to be associated with increased SD (β = 1.58; 95% CI, 0.58-2.59), COV (β = 1.19; 95% CI, 0.35-2.04), and ARV (β = 0.53; 95% CI, 0.03-1.02).

**Table 2.** Multivariable mixed effects regression on the SD, COV, and ARV of systolic blood pressure.

	SD		COV		ARV	
Variable	β (95% CI)	*P*	β (95% CI)	*P*	β (95% CI)	*P*
Age	-0.04 [-0.09 to 0.02]	.159	-0.06 [-0.10 to -0.01]	**.012**	-0.01 [-0.04 to 0.02]	.410
Hb	0.44 [0.22 to 0.67]	**<.001**	0.31 [0.12-0.50]	**.003**	0.16 [0.05 to 0.27]	**.007**
SCD						
No	0 [Ref]		0 [Ref]		0 [Ref]	
Yes	1.58 [0.58 to 2.59]	**.004**	1.19 [0.35-2.04]	**.008**	0.53 [0.03 to 1.02]	**.038**
Exercise						
Baseline	0 [Ref]		0 [Ref]		0 [Ref]	
N-Back	-0.88 [-1.06 to -0.71]	**<.001**	-0.76 [-0.91 to -0.61]	**<.001**	-0.18 [-0.23 to -0.12]	**<.001**
Stroop	-0.85 [-1.02 to -0.68]	**<.001**	-0.80 [-0.95 to -0.65]	**<.001**	-0.18 [-0.24 to -0.13]	**<.001**
Pre-PA baseline	-0.57 [-0.96 to -0.18]	**.004**	-0.51 [-0.86 to -0.17]	**.004**	-0.17 [-0.30 to -0.05]	**.006**
PA	-0.10 [-0.45 to 0.25]	.586	-0.02 [-0.33 to 0.28]	.878	0.02 [-0.09 to 0.13]	0.747
Post-PA baseline	-1.03 [-1.30 to -0.75]	**<.001**	-0.93 [-1.17 to -0.69]	**<.001**	-0.34 [-0.43 to -0.25]	**<.001**

Abbreviations: ARV: average real variability; COV: coefficient of variation; PA: pain anticipation.

Bold indicates statistically significant, *P *<* *.05.

### Changes in MAP and AI

**Figure 3.**
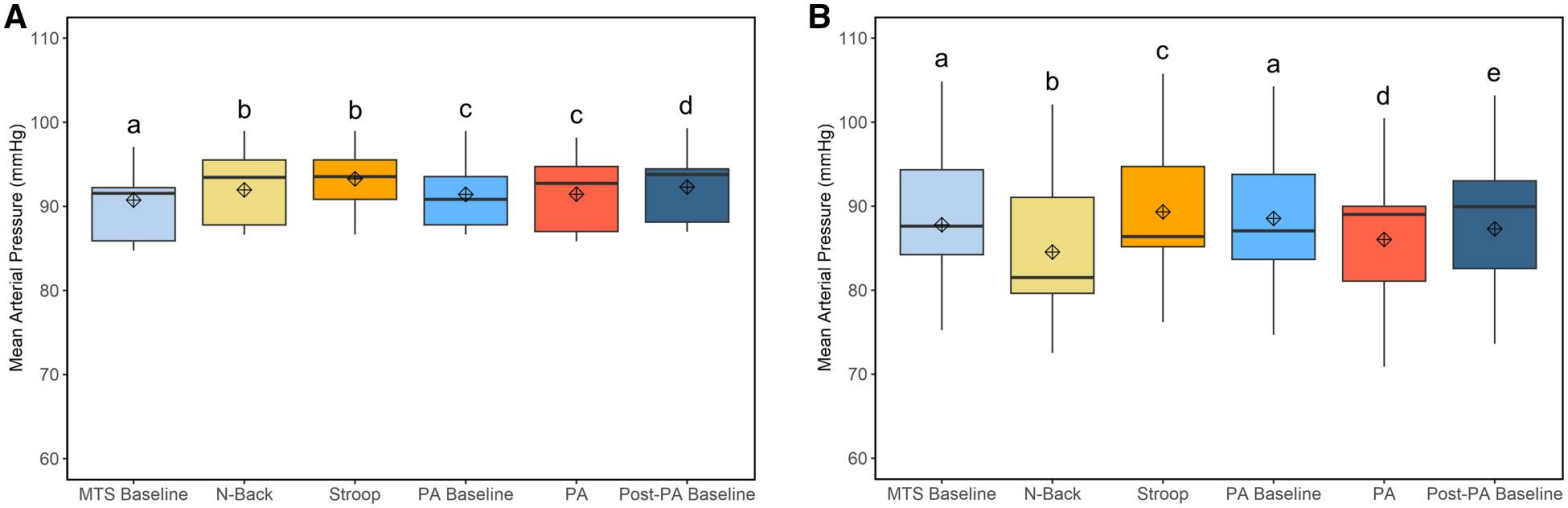
Covariate-adjusted box and whisker plot of the MAP at baseline and during study exercises for (**A**) control and (**B**) SCD patients. Increases in MAP occurred in control patients during N-back and Stroop tasks. In SCD patients, MAP significantly decreased during N-back and PA tasks relative to their respective baselines. Diamonds indicate adjusted mean values; horizontal lines indicate adjusted median values. Within each subject cohort, boxes labeled with different letters indicate a statistically significant (*P* < .05) mean difference. For example, within the control subjects in A, MTS baseline’s mean is statistically different from N-back’s mean but N-back’s mean is not statistically different from Stroop’s mean, whereas all 3 of them are different in the SCD group. MAP: mean arterial pressure; MTS: mental stress; PA: pain anticipation.

We examined the effect of stress responses on vascular modulation and blood flow by evaluating MAP and AI during the study tasks. Compared to baseline, control subjects exhibited an increase in MAP during N-back and Stroop tasks (all *P *<* *.001; Figure 3A). However, PA did not result in a significant change in MAP relative to the PA baseline for controls (*P *>* *.05). In contrast, SCD patients exhibited a significant decrease in MAP during the N-back task (*P *<* *.001), which returned to a level slightly higher than baseline during the Stroop task (*P *<* *.001; Figure 3B). Moreover, PA induced a significant decrease in MAP relative to the PA baseline in the SCD group (*P *<* *.001).

**Figure 4.**
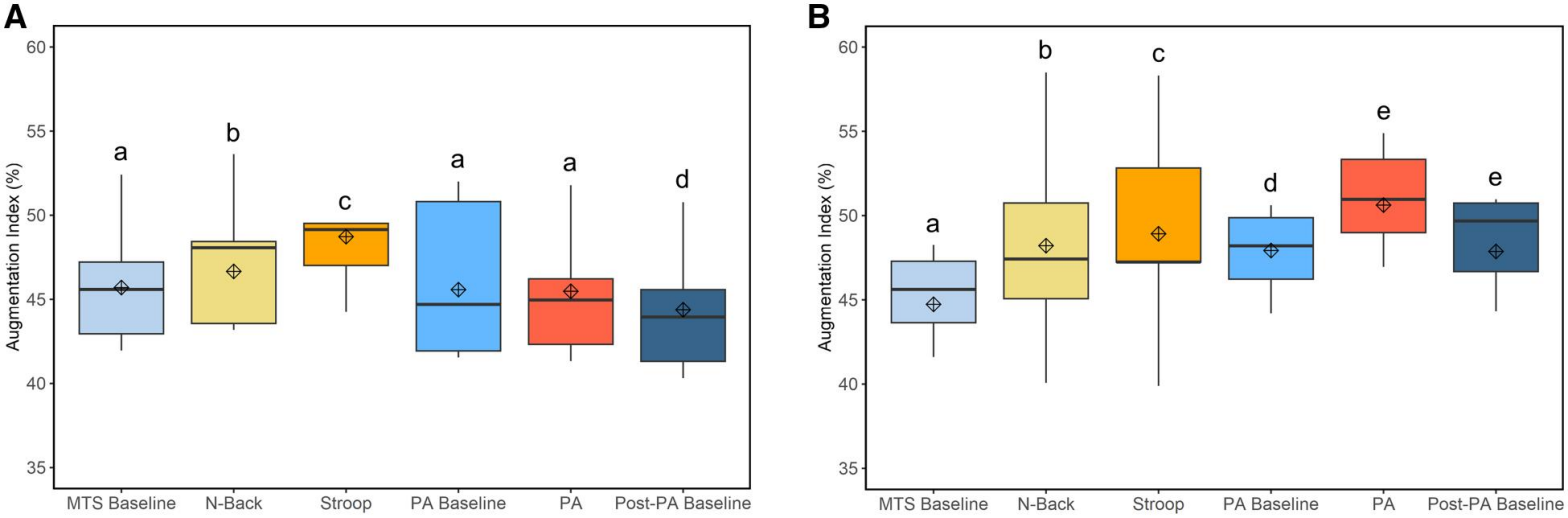
Covariate-adjusted box and whisker plot of the AI at baseline and during study exercises for (**A**) control and (**B**) SCD patients. Both groups exhibited increases in AI during N-back and Stroop tasks. AI increased during PA in SCD patients. Diamonds indicate adjusted mean values; horizontal lines indicate adjusted median values. Boxes labeled with different letters indicate a statistically significant (*P* < .05) difference in means. AI: augmentation index; MTS: mental stress; PA: pain anticipation.

Compared to baseline, both control and SCD subjects exhibited significant increases in AI following N-back and Stroop tasks (all *P *<* *.001; Figure 4). In the control group, AI returned to baseline levels following MTS tasks, which did not significantly change during PA (all *P *>* *.05). In contrast, the AI in SCD patients remained elevated at PA baseline following MTS tasks (all *P *<* *.001). Moreover, PA induced a further increase in AI in the SCD group (*P *<* *.001). Following the PA task, AI decreased to PA baseline levels, but remained elevated compared to MTS baseline (all *P *<* *.001).

## DISCUSSION

In this study, we examined fluctuations in beat-to-beat BP and the dynamic changes in arterial function in individuals with SCD, aiming to elucidate potential correlations and implications for clinical management. We identified several unique changes in BPV, MAP, and AI in SCD patients in response to MTS and PA tasks. These differences may be attributed to the unique pathophysiology of SCD, including dysautonomia and endothelial dysfunction, which can alter vascular tone and regulatory mechanisms.^47^^,^^48^ Although physical stimuli, such as pain and cold temperatures, have been studied in SCD, there are still few studies that have investigated how changes in BP, particularly very short-term BPV, and vascular tone can be induced by mental or psychological stimuli.^12^^,^^13^ Understanding this process is highly relevant to effectively managing SCD pain, as patients experience a myriad of stressors in their daily lives and can suffer higher levels of anxiety, depression, and emotional distress.^49^ Stress in SCD is characterized by ANS hyper-reactivity, with increased activation of the sympathetic nervous system and decreased parasympathetic activity.^50^^,^^51^ Autonomic dysregulation has been associated with increased BP fluctuations and clinical vaso-occlusion.^10^^,^^52^ Impaired vascular modulation and decreased regional blood flow can be particularly concerning in SCD due to an increased risk for red blood cell entrapment in small vessels. To our knowledge, this is one of the first studies to examine how beat-to-beat BPV and vascular tone are influenced by mental (MTS tasks) and psychological (PA task) stressors. We observed a distinct pattern of changes in BPV and vascular function in SCD patients, underscoring the need for holistic management strategies targeting not only the disease-specific symptoms and complications but also addressing the psychosocial aspects and stressors that impact autonomic function. Furthermore, identifying these physiological differences via quantitative measures serves as a stepping stone for future efforts aimed at predicting VOCs and mitigating pain events by implementing prophylactic interventions.^53^^,^^54^

Our multivariable analysis indicated that SCD may cause greater fluctuations in beat-to-beat BP. As the arterial baroreflex is considered an important regulator of beat-to-beat BP through its modulation of heart rate and vascular tone, our findings suggest a potential baseline impairment in the arterial baroreflex among individuals with SCD.^6^^,^^55^^,^^56^ This impairment might stem from various factors inherent to the disease’s pathophysiology. Chronic anemia and repeated VOC induce hypoxia and tissue injury, which have been shown in other clinical situations to cause impairments in baroreflex sensitivity.^57^^,^^58^ Additionally, the pro-inflammatory state characteristic of SCD may contribute to endothelial dysfunction and altered baroreflex sensitivity.^59–61^ The nitric oxide (NO) system is also involved in short-term regulation of BP. Up to 50% of patients with SCD have been reported to have endothelial dysfunction due to poor bioavailability of endogenous NO.^62^ Thus, impairment of the NO system may also contribute to higher BPV observed in SCD patients.

Unlike the control group, changes in BPV in response to MTS appeared to persist beyond the cessation of MTS stimuli in SCD patients. This prolonged response could reflect impaired ANS modulation or a slower return to baseline autonomic function in individuals with SCD. Additionally, while both groups exhibited a decrease in systolic BPV in response to MTS tasks, SCD patients demonstrated a distinct increase in systolic BPV in response to PA. This finding aligns with previous research indicating heightened ANS reactivity among SCD patients in response to physical and mental stressors.^10^^,^^50^ Additionally, some studies have shown that differences in experienced environmental stressors play a role in perceived pain intensity for individuals with SCD.^63–65^ The cumulative impact of frequent medical procedures, unexpected painful episodes, and complications related to the disease may sensitize the ANS response in SCD patients to an anticipated threat or stressor.

## CONCLUSION

Baseline measurements demonstrated a lower MAP and AI in SCD patients compared to controls. This is consistent with previous literature that reported decreased arterial stiffness and MAP at rest in SCD patients.^66^ During MTS tasks, we observed that both control and SCD subjects demonstrated increases in AI but minimal changes in MAP. This rise in AI is likely an indicator of vasoconstriction, which is known to occur during acute MTS. This stress response is a result of enhanced sympathetic modulation, which also potentially impairs endothelial function by reducing NO availability if the response is sustained. Indeed, prior reports have shown MTS tasks to decrease endothelial function, as measured by flow-mediated dilation.^67^^,^^68^ Interestingly, we observed a different pattern of changes in response to PA tasks. Following the MTS tasks, AI returned to baseline levels during the PA baseline for control subjects, but it remained elevated in SCD patients. This suggests that the vasoconstriction that occurred during MTS persisted even after the stress was removed in SCD patients. This is consistent with what was found in Veluswamy et al., which demonstrated a cumulative and progressive effect of vasoconstriction in SCD after multiple exposures to thermal pain stimuli.^69^ This persistence in vasoconstriction is important to recognize, since it makes SCD patients more vulnerable to other external inputs that could trigger VOC. The substantial increase in AI during PA in SCD patients suggests that their already elevated vasoconstriction after MTS tasks primes them for larger increases in vasoconstriction in response to PA. Even after the PA “stimulus” disappears, AI in SCD patients returned to the PA baseline but not down to the levels of the original baseline. In contrast, in control subjects, the AI became slightly lower than the original baseline level at the end of the study. Therefore, it is possible that the “cumulative effect” of vasoconstriction elicited by different types of stimuli may be what primes SCD subjects for VOC if there are subsequent events that trigger further vasoconstriction. Additionally, this observation also highlights how differences in the environmental challenges that SCD patients experience in their lives may impact their response to stressors. Although the MTS tasks cognitively challenged subjects, the PA tasks challenged them psychologically with the fear of experiencing pain. The anticipation of pain may have elicited a heightened autonomic response in SCD patients who have repeatedly endured painful VOCs throughout their lives. Therefore, in SCD management, tailored interventions focusing on stress reduction may potentially mitigate VOCs, improving overall cardiovascular health and quality of life. Further research exploring these complex interactions is necessary to guide effective management strategies for this patient population.

There are limitations in this study worth noting. Our small sample size limited the depth of our statistical analyses and did not allow for further stratification according to certain patient factors, such as hydroxyurea therapy or chronic transfusion status. Since most of our SCD patients were actively being treated and, thus, at lower risk for clinical crises, it is also possible that their responses to mental and psychological stress were not as markedly different from controls as the general SCD population would have been. Thus, a larger study that includes untreated patients may be warranted to better elucidate these differences. Additionally, although multivariable analysis was used to mitigate confounding effects, our patient sample limited our ability to delineate the effect of anemia from SCD on the autonomic regulation of vascular flow. Follow-up studies could benefit from including patients with non-SCD anemia (eg, beta thalassemia) to confirm if the vasoconstrictive properties demonstrated in our SCD subjects are similar to non-SCD patients with comparable degrees of anemia. Nonetheless, the purpose of this study was to identify any differences in how beat-to-beat BPV and endothelial function (via MAP and AI) changed in response to mental and psychological stress in SCD patients. Our findings suggest an increase in autonomic reactivity and vasoconstrictive response in SCD patients that is particularly pronounced in response to psychological stressors, which holds potential for predictive and potentially preventive pain measures.

## Supplementary Material

yoae010_Supplementary_Data
