# Supplementary material for: Beat-to-beat analysis of hemodynamic response to mental and psychological stress in sickle cell anemia
Source: J Sick Cell Dis. 2024 Oct 28;1(1):yoae010. doi: 10.1093/jscdis/yoae010 (PMC11951424; doi:10.1093/jscdis/yoae010)
Supplement: yoae010_Supplementary_Data [file yoae010_Supplementary_Data.zip › Supplementary Material.docx]

Supplementary Materials

Beat-to-beat analysis of hemodynamic response to mental and psychological stress in sickle cell anemia

Arash Abiri, Sara Marmarchinia, Payal Shah, Wanwara Thuptimdang, Thomas D. Coates, Michael C.K. Khoo, Michelle Khine


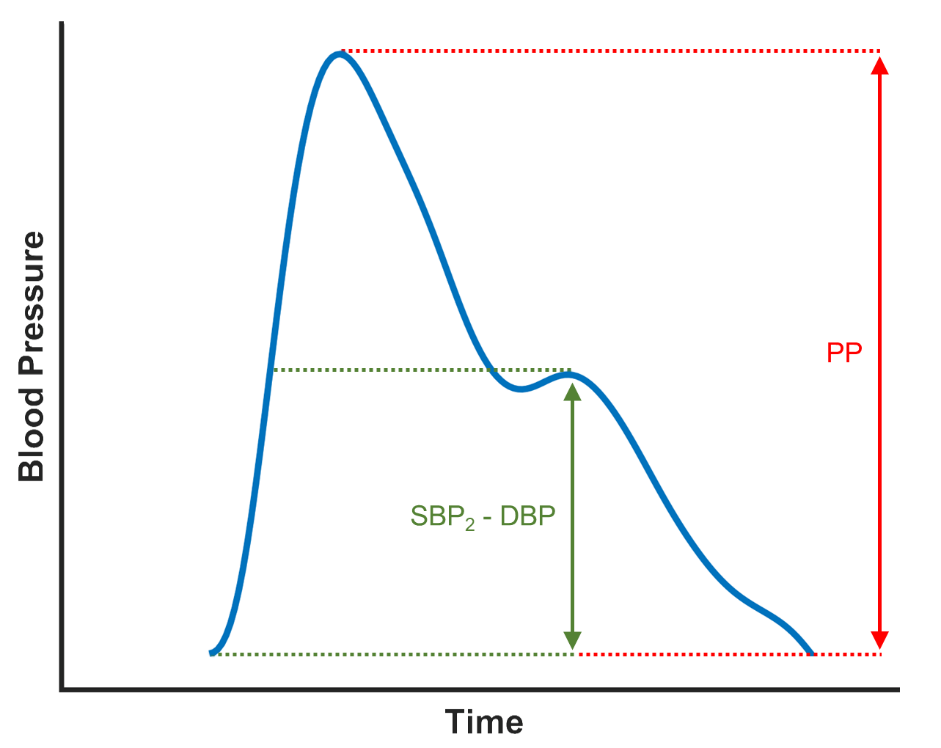


**Figure S1.** Plot of a representative blood pressure waveform (blue line). Peripheral augmentation index was calculated by dividing the difference of the late systolic peak (SBP_2_) and diastolic blood pressure (DBP) by the pulse pressure (PP).


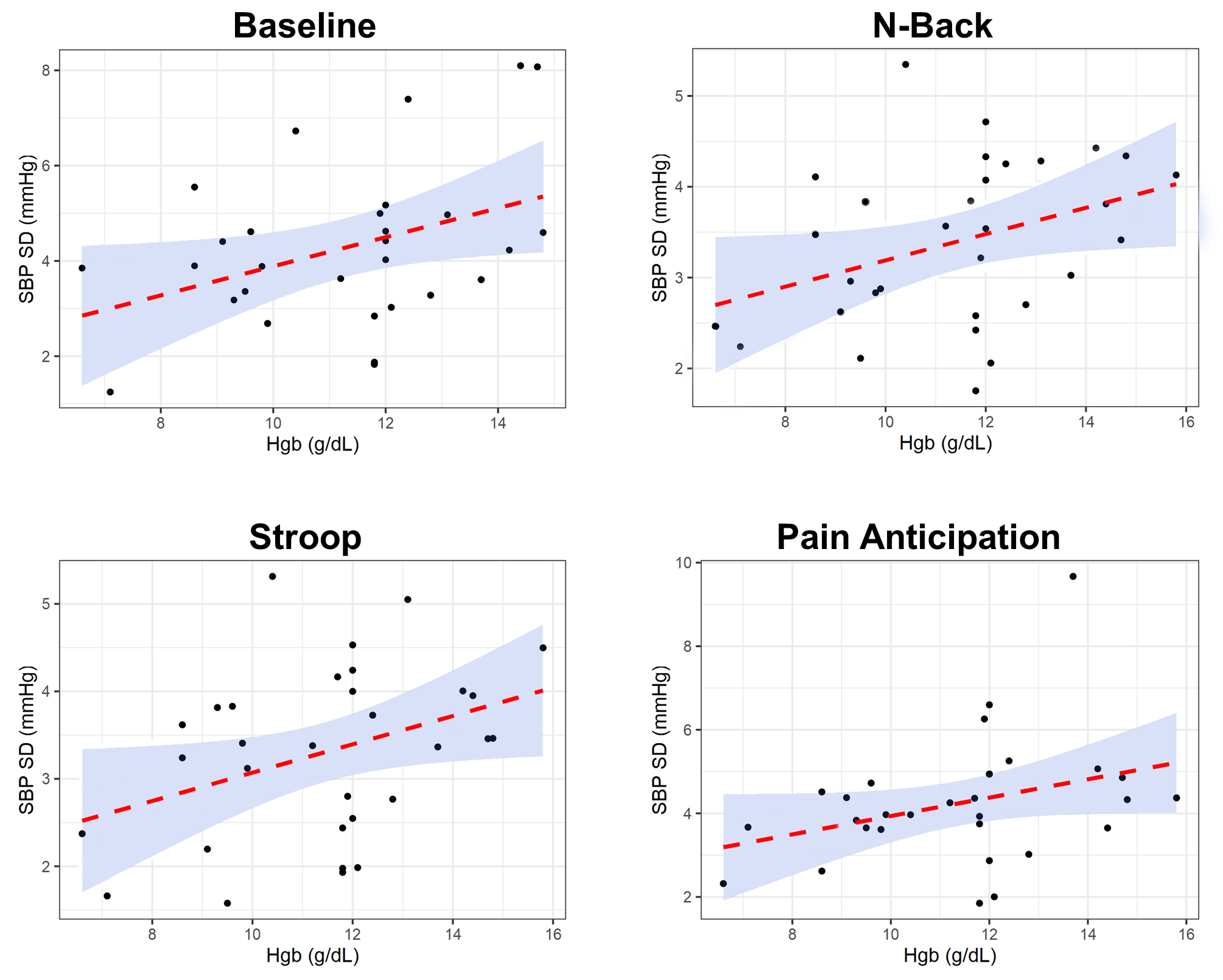


**Figure S2.** Plots of the standard deviation (SD) of beat-to-beat systolic blood pressure (SBP) versus hemoglobin (Hgb) for all subjects stratified by task There was a significant linear relationship between SBP SD and Hgb during baseline (*p*=0.035, R^2^=0.154), N-back (*p*=0.043, R^2^=0.133), and Stroop (*p*=0.039, R^2^=0.138) tasks, but not during the pain anticipation (*p*=0.072, R^2^=0.111) task. Red dotted line represents the linear regression. Blue shaded region indicates the 95% confidence interval of the regression model.


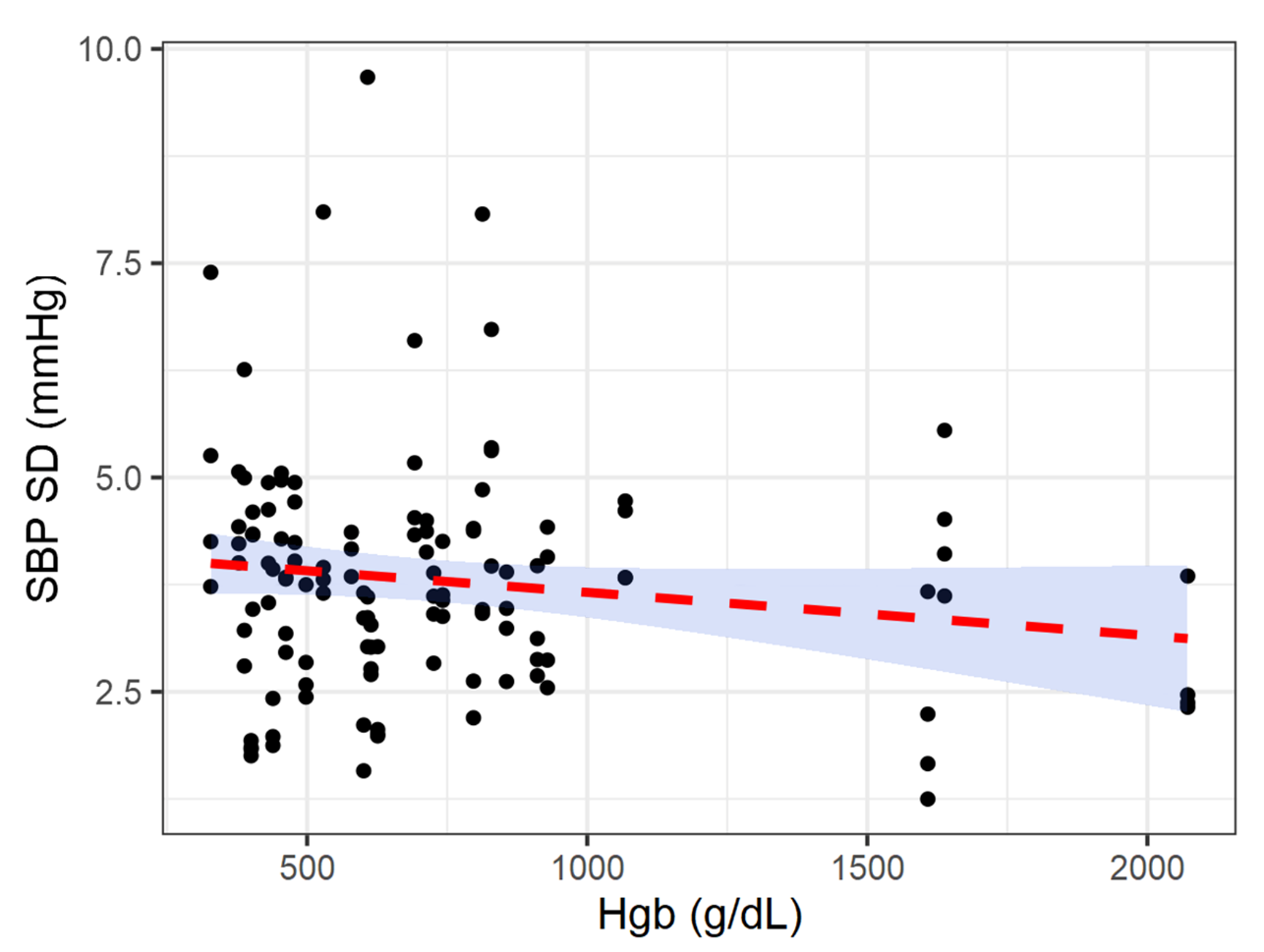


**Figure S3.** Plot of the standard deviation (SD) of beat-to-beat systolic blood pressure (SBP) versus hemoglobin (Hgb) for all subjects. There was no significant linear relationship between SBP SD and Hgb (*p*=0.107; R^2^=0.013). Red dotted line represents the linear regression. Blue shaded region indicates the 95% confidence interval of the regression model.
