# Supplementary figures and images for: Beat-to-beat analysis of hemodynamic response to mental and psychological stress in sickle cell anemia
Source: J Sick Cell Dis. 2024 Oct 28;1(1):yoae010. doi: 10.1093/jscdis/yoae010 (PMC11951424; doi:10.1093/jscdis/yoae010)

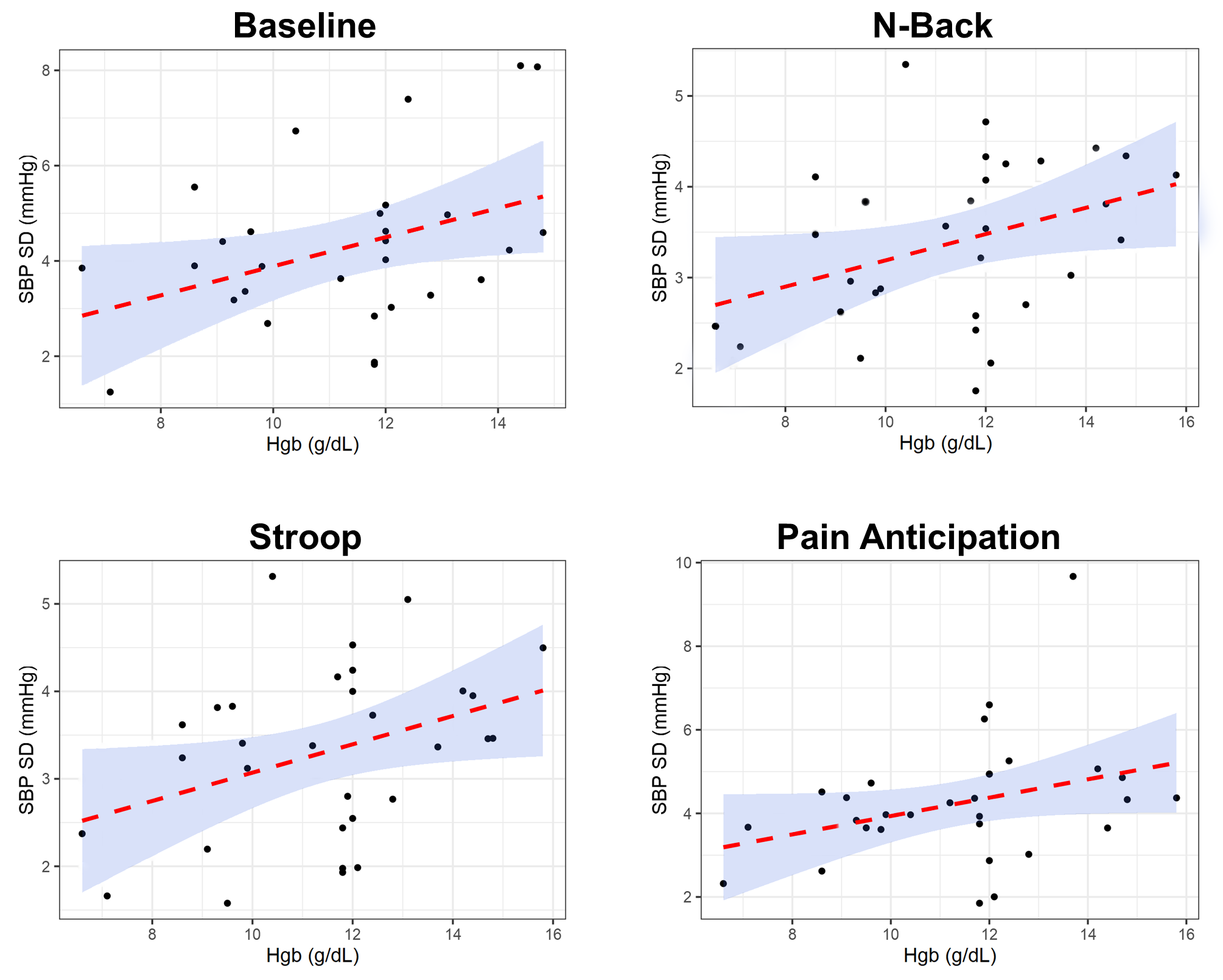

Supplement: yoae010_Supplementary_Data [file yoae010_Supplementary_Data.zip › Figure S2.tif]

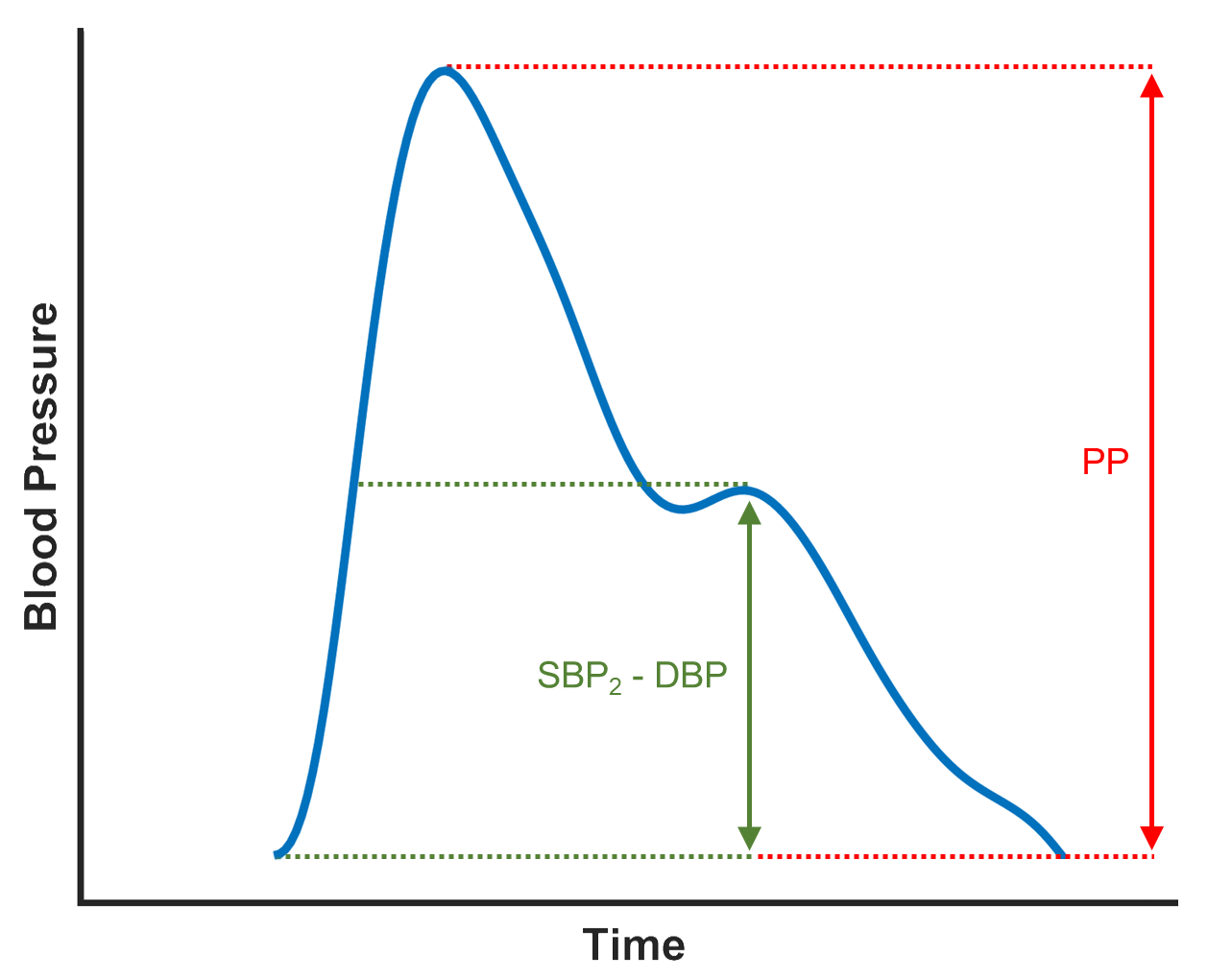

Supplement: yoae010_Supplementary_Data [file yoae010_Supplementary_Data.zip › Figure S1.tif]

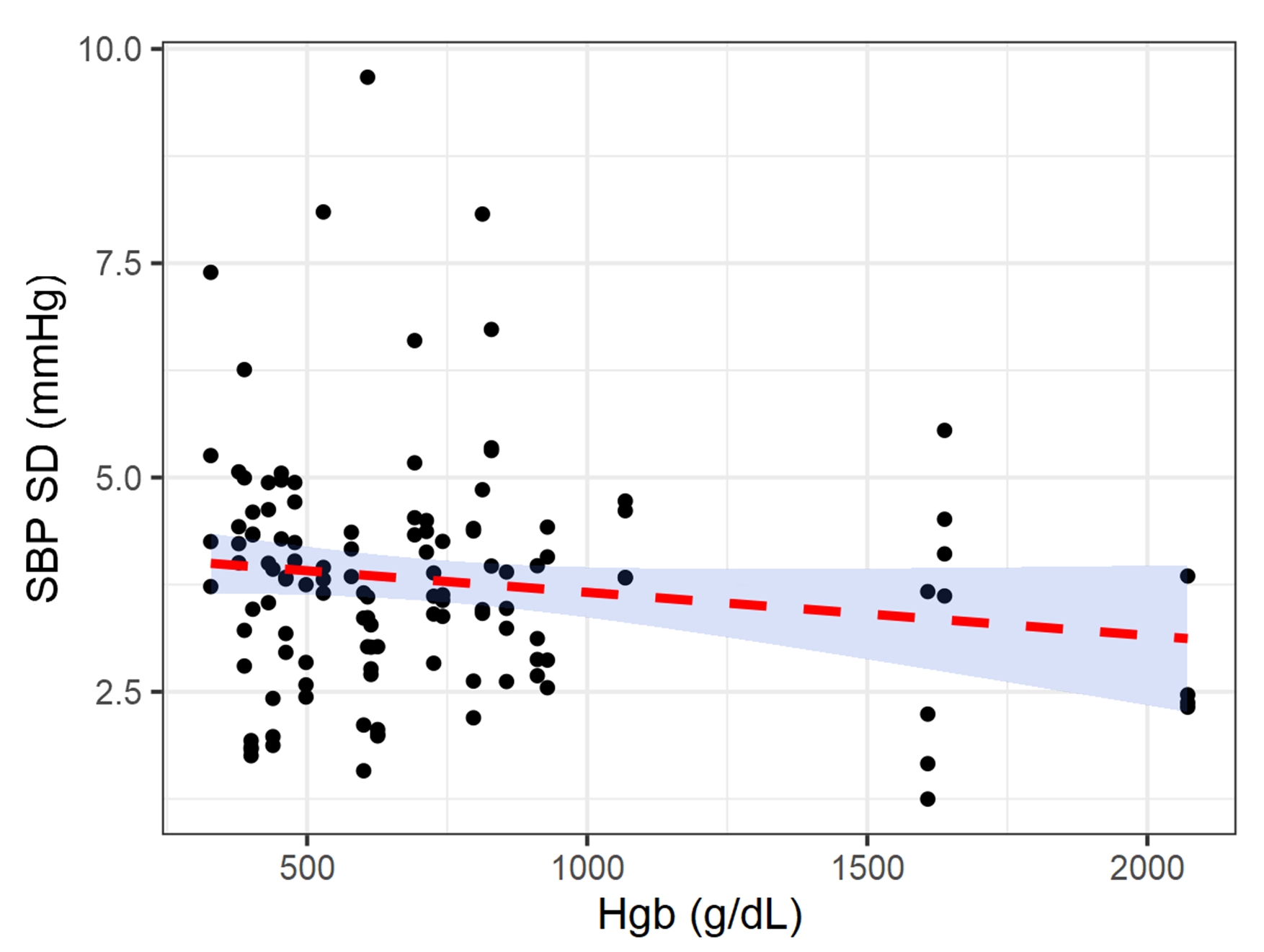

Supplement: yoae010_Supplementary_Data [file yoae010_Supplementary_Data.zip › Figure S3.tif]
